# “Smartphone Apps Are Cool, But Do They Help Me?”: A Qualitative Interview Study of Adolescents’ Perspectives on Using Smartphone Interventions to Manage Nonsuicidal Self-Injury

**DOI:** 10.3390/ijerph18063289

**Published:** 2021-03-22

**Authors:** Anja Čuš, Julian Edbrooke-Childs, Susanne Ohmann, Paul L. Plener, Türkan Akkaya-Kalayci

**Affiliations:** 1 Department of Child and Adolescent Psychiatry, Medical University of Vienna, 1090 Vienna, Austria; anja.cus@meduniwien.ac.at (A.Č.); susanne.ohmann@meduniwien.ac.at (S.O.); paul.plener@meduniwien.ac.at (P.L.P.); 2 Evidence-Based Practice Unit, University College London, London WC1E 6BT, UK; Julian.Childs@annafreud.org; 3 Anna Freud National Centre for Children and Families, London N1 9JH, UK; 4 Department of Child and Adolescent Psychiatry and Psychotherapy, Ulm University, 89081 Ulm, Germany

**Keywords:** NSSI, mHealth, mental health, smartphone apps, adolescence, qualitative study, user perspectives, user engagement, design framework, design implications

## Abstract

Nonsuicidal self-injury (NSSI) is a major mental health problem associated with negative psychosocial outcomes and it most often starts in early adolescence. Despite this, adolescents are rarely involved in informing the development of interventions designed to address their mental health problems. This study aimed to (1) assess adolescents’ needs and preferences about future interventions that are delivered through smartphones and (2) develop a framework with implications for designing engaging digital mental health interventions. Fifteen adolescent girls, aged 12–18 years, who met diagnostic criteria for a current NSSI disorder and were in contact with mental health services, participated in semi-structured interviews. Following a reflexive thematic analysis approach, this study identified two main themes: (1) Experiences of NSSI (depicts the needs of young people related to their everyday experiences of managing NSSI) and (2) App in Context (portrays preferences of young people about smartphone interventions and reflects adolescents’ views on how technology itself can improve or hinder engaging with these interventions). Adolescent patients expressed interest in using smartphone mental health interventions if they recognize them as helpful, relevant for their life situation and easy to use. The developed framework suggests that digital mental health interventions are embedded in three contexts (i.e., person using the intervention, mental health condition, and technology-related factors) which together need to inform the development of engaging digital resources. To achieve this, the cooperation among people with lived experience, mental health experts, and human computer interaction professionals is vital.

## 1. Introduction

Nonsuicidal self-injury (NSSI), defined as deliberate destruction of body tissue in *absence* of suicidal intent, is an important mental health concern worldwide [1]. Approximately 18% of adolescents (age range 11–18 years) report at least one life-time event of NSSI [2]. NSSI usually starts in adolescence, most often between the age of 12 and 14 [3] and is a risk factor for suicidal behavior [4]. A recent meta-analysis showed that people engage in NSSI predominantly for intrapersonal reasons, such as emotion regulation but also for interpersonal reasons, such as communicating distress [5]. Despite the poor psychosocial outcomes connected with NSSI, young people rarely seek help from health professionals [6,7]. Additionally, adolescents are often not aware of the available help, believe they should be able to cope with NSSI by themselves or do not seek help to prevent hurting people near to them and to avoid being labelled as *attention seekers* [6,8]. These barriers to help seeking could be partially addressed by directing young people to technology-enabled resources. The onset of the COVID 19 pandemic has further highlighted the importance of using technology to deliver mental health support [9].

However, seeking help online can be described as a *double-edged sword*: for example, through using social media to discuss and view NSSI, young people may feel supported and understood, but can also feel motivated to further engage in NSSI [10,11]. Clearly, while young people search for help online, it is important to consider how to meet their needs and at the same time pay attention to the potential risks.

Digital interventions, especially those delivered by means of mobile phones, hold promise to overcome barriers to treatment and provide support regardless of the user’s location [12]. This type of support may be especially relevant for young people who worldwide increasingly own and use mobile devices [13]. However, a recent content analysis of available NSSI apps showed that only a few apps that are freely available online were developed by a trusted source [14] and there are very few, that reported data from randomized controlled trials [15,16]. The lack of evidence-base is the most commonly recognized shortcoming of existing digital interventions [17], and app evaluation models are being developed to address this issue [18].

Evidence-base for digital interventions that focus primarily on NSSI is scarce and two studies have reviewed such interventions in the broader context of self-injurious behaviours. A systematic review of online interventions to reduce self-harm (i.e., self-injury *with* or *without* suicidal intent) and suicidal ideation found that in general the use of online interventions led to a decrease in suicide ideation, but it had no impact on self-harm [19]. Another review identified four trials related specifically to NSSI [20], and while these studies reported reduction in NSSI, their findings need to be interpreted with caution due to lack of control groups [21,22], absence of maintained effect in a follow up study [16] and absence of effect when compared to active control groups [15]. From the trials that focused on either NSSI or self-harm only two were developed for adolescents and out of these two only one was delivered by smartphone [20,21,23].

In addition to low evidence-base in smartphone interventions for NSSI, a further shortcoming of technology-enabled interventions is disengagement with the developed interventions [24]. For example, a systematic review of digital interventions for self-harm suggested that using gamification approaches in developed interventions may not be sufficient to sustain engagement with them [19]. From the perspective of human computer interaction (HCI) and implementation science, it is important to learn about and take into account users’ needs and preferences in order to improve patients’ clinical outcomes [25,26]. Furthermore, strategies that have been proposed to increase engagement with mental health interventions include *interactive* experience with technology, *personal* experience that responds to user’s needs, *supportive* elements such as guidance within technology and *social* strategy that allows contact with other people [26].

Although some exceptions exist [27], research that focuses on understanding adolescents’ needs and preferences in the context of digital mental health interventions is very rare [28]. Adolescents from community samples, on the other hand, wish for interventions that are relevant, easy to use, allow contact with peers, provide confidentiality, and give control and choice over using the mobile health (mHealth) interventions [29]. Young people have also reported diverse preferences for digital mental health interventions that need to be outlined and addressed to improve engagement with digital therapies [30].

To our best knowledge, no study has qualitatively assessed perspectives of young patients who engage in NSSI on apps, although involving users in the research and intervention development process is the time when meaningful changes are still possible [31]. Thus, the aim of this study is to explore the needs of adolescents with lived experience of mental health condition, namely NSSI, and use this knowledge to develop a framework for designing engaging smartphone interventions for mental health.

## 2. Materials and Methods

This study employs a qualitative approach because it is most suitable to capture people’s experiences and perspectives [32].

### 2.1. Recruitment

Our sample was based on a purposive sampling approach, consisting of adolescents who were in contact with mental health services due to recent repetitive NSSI. We estimated such sample would have adequate experiences to provide insight to our research questions. Psychiatrists at a Department of Child and Adolescent Psychiatry informed their eligible patients about the study. Patients were eligible to participate if they fulfilled the DSM-5 Section 3 criterion of engaging in NSSI at least five times in the last year [33] and were fluent in either German or English. In line with the DSM-5 criteria, patients were not eligible to participate if they engaged in subthreshold NSSI or NSSI as part of developmental disorders [33]. Due to ethical considerations, severe suicidal ideation was also an additional exclusion criterion. Upon expressing the interest to participate, patients and their parents were contacted by the third author and informed about the study’s aim and procedure, including audio-recording the interview, the voluntary nature of the participation and that their decision to (not) participate will not have any influence on the treatment they receive.

Sixteen participants were interviewed. One interview (P6) was excluded from the analysis due to reported one event of self-injury and thus not meeting DSM-5 diagnostic criteria for NSSI. The final sample thus consisted of fifteen female adolescents aged 12 to 18 years (*M* = 15.2; *SD* = 1.61). Male participants were not excluded intentionally, only one male participant agreed to be interviewed but he withdrew consent for unknown reasons before the interview would take place.

### 2.2. Data Collection

Interviews are commonly used to include active user participation in user-centred systems and were thus utilized to collect data [34]. The semi-structured interviews were conducted between June 2018 and June 2019 by the third and/or first author in a therapist’s office without significant interruptions. Both interviewers were not involved in the treatment of these patients prior or at the time of the interviews. The third author (psychologist with additional psychotherapeutic education) was present at all interviews to provide psychological support if needed. This option was not used, and participants reported that they felt either good or neutral during and after the interviews.

The interview topic guide was developed based on the guidelines to conduct interviews [35,36], theory- and practice-based understanding of NSSI and our research questions. The open questions in the interview revolved around the following topics: (1) Experience with NSSI, (2) Managing NSSI, (3) Relationship between technology and NSSI, (4) Envisioning a mobile app for their NSSI management, (5) Opinion on future smartphone-delivered interventions facilitated by prompt questions and interfaces. An average interview lasted 40 min; one was conducted in English and the rest in German. All interviews were audio-recorded, transcribed verbatim in the language in which they were conducted and anonymized in the process of transcription. To maintain consistent approach in coding, all interviews were coded in German. After the analysis with German codes and themes was completed, the first author translated them together with their illustrative quotations. Two native German speakers independently checked these translations to ensure consistency of meanings.

### 2.3. Data Analysis

The data was analysed using reflexive thematic analysis by combining both constructivist and experiential approaches [32,35]. Thematic analysis is commonly used in research on people’s opinion, experiences and needs. We followed Braun and Clarke’s [28] six-step guide that involves (1) familiarizing oneself with the data, (2) generating initial codes, (3) searching for themes, (4) reviewing themes, (5) defining and naming themes, and (6) producing the report. To illustrate, the analysis started with the first author listening to the audio-recordings of the interviews and reading the transcripts several times to reach data familiarization. Next, transcripts were uploaded in the qualitative software program Atlas.ti version 8 (ATLAS.ti Scientific Software Development GmbH: Berlin, Germany) to assist the analysis. In this program, the first author assigned initial codes and regularly discussed the coding process with the fourth author and in addition consulted with the third author (i.e., the second interviewer) whenever the meaning of participants’ replies was unclear. The analysis was an iterative process, whereby codes were crosschecked and when necessary revised. Through this process, the codes became less descriptive and more interpretive. Analytic ideas and identified patterns were discussed within a research team to follow the guide steps three to five. This process involved going back to the data, re-reading the transcripts and shaping candidate themes based on the patterned responses. The themes and subthemes were developed inductively from the data and were not determined in advance. Agreement within the team on data saturation (i.e., when additional interviews did not produce new knowledge) was reached after 15 interviews. Following the qualitative paradigm that estimates trustworthiness to be a better justified quality criterion as opposed to reliability, we strived to achieve trustworthiness through following the Brown and Clarke’s checklist of quality criteria for thematic analysis [35] (p. 287).

In addition, we conducted a follow-up interview with one participant (P9) as part of a member checking technique to increase interpretive validity and ensure the analysis is credible from the participants’ point of view [35]. The analysis was further shaped through the last step, i.e., the writeup process. The writing and analysis are usually intertwined in qualitative research [35]. This study is reported in line with the COREQ consolidated criteria for qualitative research [37].

## 3. Results

The primary clinical diagnoses of the participants (*n* = 15) based on the ICD 10 criteria and assigned by board certified child and adolescent psychiatrists were: adjustment disorder (27%), major depressive disorder (27%), acute stress reaction (13%), attention-deficit hyperactivity disorder (13%), borderline personality disorder (13%), and bulimia nervosa (7%). Comorbid disorders were frequent and included major depressive disorder (33%), post-traumatic stress disorder (PTSD) (27%), borderline personality disorder (20%), attention-deficit hyperactivity disorder (20%), cannabis abuse (13%), social phobia (13%), agoraphobia (7%), and alcohol abuse (7%).

**Figure 1:**
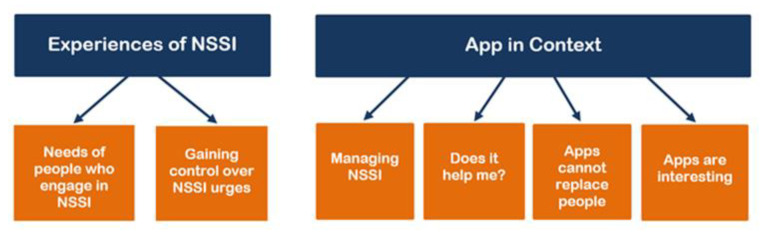
Thematic map representing themes and their subthemes. (NSSI = Nonsuicidal self-injury).

The patterns in this study were collated into two central themes: *Experiences of NSSI* and *App in Context* that together consist of six subthemes (Figure 1). The following paragraphs present subthemes together with illustrative quotations that were translated from German.

### 3.1. Experiences of NSSI

This theme describes adolescents’ needs related to NSSI and their coping strategies.

#### 3.1.1. The Needs of People Who Engage in NSSI

Participants described that they struggle with overwhelming thoughts and emotions and with NSSI. Shortly before engaging in NSSI behaviour, they often experienced unpleasant emotions and/or felt helpless. Unpleasant thoughts were mostly directed towards themselves in which case the NSSI had self-punishing qualities.

P14: *“Ok. So, it mostly arises from the situation that triggered that, are the situations where I say: ‘Yes, you are a disappointment’, so I say it to myself, that I am a disappointment and need to be punished for that, so ‘for this mistake you need to be punished’ and I accept that. And well, that is how it is and then I did it.”*

Participants’ accounts of NSSI were often ambivalent as indicated through participants talking about both the helpful aspects of NSSI as well as its unpleasant aspects. The helpful aspects included feeling free of thoughts and emotions or feeling stable again. To the contrary, a few of them experienced feelings of guilt after engaging in NSSI or noted that it *works* only temporarily. Further negative outcomes of engaging in NSSI involved receiving invalidating reactions from their surroundings.

P10: *“I mean obviously, like covering scars and like when people see scars like they get freaked out and they start asking questions, so that’s obviously something that I try to avoid.”*

Participants reported that they experience the *urge* to engage in NSSI and two of them compared this urge to addiction. Participants referred to NSSI as something that comes in waves, whereby its intensity and frequency changes depending on stress and life situations. However, one participant described it as a routine and another one as a part of her life.

P13: *“For me it simply became a part of my life/…/. It is also difficult to stop with it, as it is so much inside of my everyday life.”*

Although a few participants learned to signal to other people that they need support, most of them said they struggle with reaching out for help.

I: *“And what do you need, when you have the feeling you want to injure yourself?”*

P5: *“Simply to talk, but I have the problem for example, that I cannot say, yes, ‘Hi, I need help, I need someone to talk to.’”*

#### 3.1.2. Gaining Control over NSSI Urges

Coping with NSSI urges and thoughts ranged from having the feeling of no control over NSSI to successfully implementing (mostly dialectical behavioural therapy—DBT) skills.

P11: *“I do the opposite. I learned that in [the DBT] skills group. I do the opposite of the feeling that I have. For example, if I am sad and feel like crying, I wipe my tears and put a smile on my face.”*

I: *“How was that before the skills group?”*

P11: *“Then I would cut myself.”*

Some participants were successful in resisting NSSI through thinking that this behaviour would hurt people around them. Other coping strategies included distracting themselves through chatting with friends online or in person, watching videos on their smartphones or intentionally seeking company to reduce the risk of NSSI. Participants also found it helpful to be in therapy where they can talk without feeling judged or receive medications. Listening to music was helpful as well but they noted that it can also lead to a negative affective outcome. Reaching out was not always productive, one participant shared an invalidating experience when calling a hotline:

P9: *“I called a hotline and that did not go well/…/And then she repeated all the time that life is beautiful. And then I hang up.”*

I: *“Ok. And when you called, what would help you more than someone who says that life is beautiful?”*

P9: *“[To hear] that life can be absolutely crap.”*

While participants often used their phones to connect with others, distract themselves and lift their mood, the majority did not use the Internet or telephone with the intention to reduce NSSI. Two participants also reported using the Internet in a counterproductive manner through exchanging tips on how to self-injure. Participants also noted different experiences with managing NSSI. Some of them never tried to prevent NSSI or described NSSI as *too fast* to find a solution, other reported not engaging in NSSI for a longer while and then returning to it. Additionally, their well-developed coping strategies were sometimes not available or despite being available, they nevertheless engaged in NSSI.

P10: “So, I mean, I think letting out like your anger in art room doesn’t really work because it’s not as satisfying as like cutting or whatever; but the whole thing is that you need to know that it’s bad for you and that it like it hurts people around you and I mean, yeah, that is my answer.”

Coping strategies changed over time and were also facilitated or hindered by environment. Most participants recognized that if they were alone, they were more likely to engage in NSSI while in school it was harder to distract themselves from NSSI urges.

I: *Do you try to distract yourself from self-injuring?*

P3: *Often.*

I: *How do you do this?*

P3: *It depends on where I am./…/At home drawing, music, series or a book. In school, nothing works for me anyways*.

### 3.2. App in Context

This theme refers to young people’s preferences for future digital interventions.

#### 3.2.1. Managing NSSI

When feeling the urge to engage in NSSI, participants reported the inability to think clearly and not being able to read large chunks of text. That is why they felt that interventions should offer a complete distraction or help in reaching different thoughts.

P9: *“So, with me it is so, I cannot think clearly in the situation, to solve problems is then not even an option, it is the feeling of not being able to do anything, that leads to it.”*

Support that they wish to receive needs to be specific for NSSI and that includes support during an acute state of mind.

P14: *“If there would be a button that can do it—to help immediately. That would be for me a total rescue./…/It also depends on umm the degree, how severely one is affected by self-injury. There are some, who can resist it and some who really cannot resist it./…/And that one would before it comes that far, that one would get an immediate help.”*

Participants also expressed the wish to track the absence of NSSI, their emotional state, and helpfulness of mHealth exercises. Those with experience in dialectical behavioural therapy (DBT) suggested to have ”DBT skills” displayed in an app. They further noted that they want to feel supported through an app at diverse time points, e.g., before NSSI, after an NSSI act and even after they in general stop engaging in NSSI. Instead of learning about NSSI in general, participants want to learn about alternatives to NSSI and understand why they engage in it.

Participants often expressed the wish to talk with other people through future NSSI apps to learn what is most helpful. Their wish to connect included talking with therapists or with people with past and/or present NSSI experience. They also noted that these exchanges should be moderated because talking with peers can also trigger NSSI.

I: *“Do you have another idea, about an app?”*

P2: *“Well, perhaps so that you can get into contact with others, can somehow talk, because there often are unfamiliar people, with unfamiliar people it is often easier to talk compared to those that know you, those with whom you are together the whole day. Perhaps like a chat, so that a lot of people can write with each other, like a group.”*

I: *“Ok. And who would be the people, are these your peers or older or adults?”*

P2: *“Well, adults that have a clue about it, that how I…”*

I: *“So, professionals.”*

P2: *“Yes, rather professionals.”*

I: *“So not peers, but…”*

P2: *“No, not that, because that can go wrong, you never know.”*

#### 3.2.2. Does It Help Me?

This subtheme refers to the wish of young people to personalize interventions and make them more relatable to their experience. To illustrate, participants want to insert their own content in the app and align it with their interests and preferences.

P11: *“What would I insert into it? That you first know when you the whole time only feel so so sad, that you know why are you sad./…/I would insert something about me inside. For example, what is my name and how I live. No clue, I do not know, but first tell a bit about myself.”*

Another recurrent aspect was having a *choice* whether to engage in an intervention and how much time to spend on it. Furthermore, when reflecting on possible content (e.g., breathing exercises), participants noted that some strategies are just not for them and that although ideas might be good generally, they would not be helpful for them. Observing that intervention helps them or that they feel better after using it, was the most prevalent reported motivation to engage with the intervention.

I: *“Which rewards should an app have that it keeps you motivated to use it?”*

P8: *“Only that it helps, that is actually the only thing.”*

Other personal preferences included developing interventions that are meaningful, relevant to their experience, age-appropriate and use language that is not too professional.

#### 3.2.3. Apps Cannot Replace People

This subtheme portrays remarks of young people that involve generally unfavorable aspects of using technology as well as critical remarks for the use of technology in the NSSI management.

Potential troubles in using technology as identified by the patients included having to secure access to the Internet to run an intervention, having an app that needs a lot of time to load and apps that take a lot of storage place on the phone. Strategies or exercises that required a lot of effort from them were perceived as a hurdle. Participants expressed data management and security concerns, such as the possibility to lose the inserted data and not knowing who might use their data. Password protection was desired especially when they are expected to upload personal content.

One participant expressed that in apps there is no one on the other side who would actively listen to you, and some perceived knowing that a person talks with a chatbot as a potential obstacle.

P14: *“So, I know many apps, I don’t know, how my app would look like, but from what I’ve seen, there are these AI, these fake chats, where one can write into and one can talk with a robot, so that one does not feel alone or something like that. It can be good for some people, but for the others, it can be quite disappointing to notice, that one does not talk with a person. So, it has a different feeling to it. One knows exactly what is human and what is not human.”*

Additionally, a few participants suggested that people should talk with other people instead of using technology to manage NSSI.

I: *“How do you use the Internet to feel better?”*

P15: *“/…/At the moment not at all, because I prefer to talk with people who I know, to talk about it in person. Mostly I then meet my friends and talk with them.”*

Participants reported having problem envisioning an appealing format or relevant content of the future interventions, also because they were not aware what is possible to implement in a digital format. Finally, one participant remarked that sometimes one loses interest in an app without a particular reason.

#### 3.2.4. Apps Are Interesting

This subtheme revolves around stated advantages of using technology and preferences for the future interventions’ format.

An attractive aspect of using apps to manage NSSI is novelty, expressed through participants’ wish to regularly see new content. Accessibility and convenience of using smartphones to manage NSSI was another recognized asset.

P10: *“Yeah, I mean, your phone, definitely I mean, I guess everyone has like their music on their phone and like games or different chats or like social media, so, yeah, so I think that’s something that is like since it’s always like attached to you, you can use it to actually benefit you in that way.”*

Another exciting possibility included the use of gamification elements, such as being presented with challenges, collecting badges, taking care of a character and unlocking new content through using an app.

P1: *“It could be like a game. So that when you… Or one should take a photo of their hand if they injured themselves or not. And you log that in. Like a photo album. Yes. Like a photo album where you take a photo of your hand every day whether you injured yourself or not. And then at the end of the month you receive an award or so. Or, I don’t know, a voucher or something like that.”*

Participants were interested in using formats that are visually appealing, have a clear structure, are simplified, easy to use and are logically divided into categories. Examples of possible presenting formats included displaying tips to manage NSSI, videos or short clips. Interestingly, a couple of participants noted that they already searched for an app that could help them and they expressed openness towards using apps for NSSI.

P2: *“I cannot imagine it [how it would look like]. I found the idea interesting, that is why I do this [the interview]. It would be cool if there is something like that.”*

P13: *“Helpful, actually [referring to the interview experience]. So, I find this with the apps cool, that you think about some things and also reflect again about these strategies, if you could use them.”*

### 3.3. Framework for Designing Digital Mental Health Interventions Derived from the Context of NSSI

**Figure 2:**
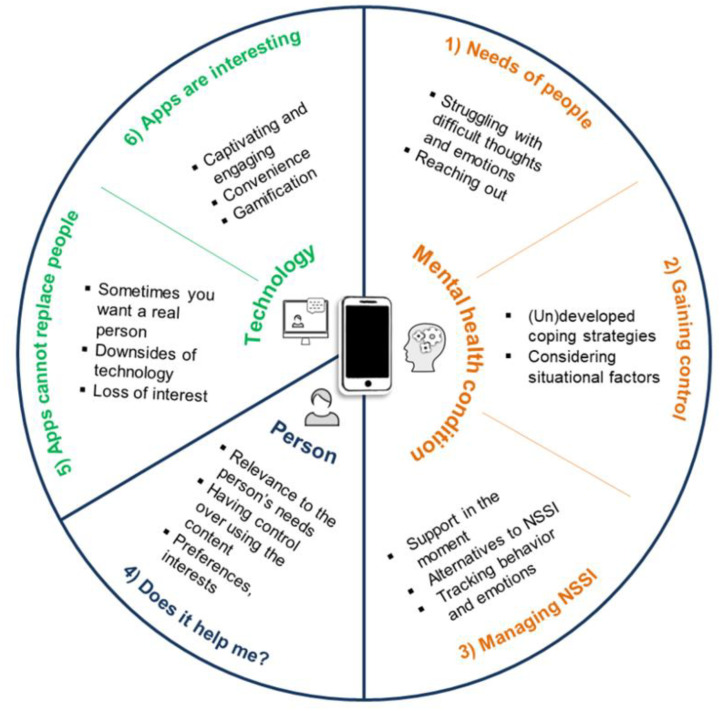
Framework for designing engaging digital mental health interventions derived from the context of NSSI.

This paragraph graphically summarizes the main content of the six subthemes and displays them along the three contexts that may influence engagement with the future interventions: mental health condition, person and technology (Figure 2). We use this graphical summary as a scaffold for generating ideas for designing future interventions (see *Design Implications for Digital Interventions*).

## 4. Discussion

### 4.1. General Discussion

Using a qualitative thematic analysis approach, this study identified young people’s needs and preferences in regard to digital support for NSSI management. The first theme *Experiences of NSSI* facilitates envisioning the everyday context in which young people will use digital interventions and through this it identifies areas in which apps could support them. Consistent with previous research on NSSI, the first theme reveals that participants struggle with difficult thoughts and emotions, self-hatred and reaching for help [5].

The subtheme *Gaining Control over NSSI Urges* describes diverse coping behaviors of young people including that different strategies work in school compared to home. Research to date has similarly identified that young people who engage in NSSI often use coping strategies that make them more vulnerable to further engage in NSSI [38]. This is an important finding, because for the interventions to be meaningfully implemented they will not only need to fit into the daily life of young people but will also need to deliver interventions that support adaptive coping strategies.

The second theme *App in Context* reveals preferences of young people in regard to digital interventions. This theme shows that future interventions need to address young people’s mental health condition (subtheme *Managing NSSI*), be perceived as helpful and aligned with the person’s preferences (*Does it help me?*) and use the favorable aspects of technology and minimize the frustrating aspects (*Apps are interesting* and *Apps cannot replace people*). In terms of specific intervention ideas, our participants wished for distraction and immediate support in the moments of crisis; such ideas are consistent with previous research on digital interventions for self-harm [27].

Similarly, a qualitative study with young people who used an app to reduce self-harm, identified that participants appreciated aspects such as personalization, ease of use, relevance, privacy, and helpfulness of the developed app [39]. Thus, our findings suggest that young people who engage in NSSI share similar preferences compared to those who engage in self-harm (i.e., self-injury regardless of suicidal intent). Furthermore, identified preferences were also similar to those from community samples [29], but the topics of helpfulness and support in crisis moments seem especially important to young people with mental health problems.

Conflicting preferences about the extent of communication, visual appeal and gamification elements observed in our study were also described by other researchers [30] and suggest importance of developing digital resources that respond to diverse needs among similar target groups. Finally, participants expressed openness and interest to use digital tools to manage NSSI.

### 4.2. Design Implications for Digital Interventions

Current digital interventions struggle with low *real-world uptake* [28]. For this reason, we present our findings in the form of a framework in order to generate ideas on how future mental health interventions could potentially be more engaging. We portray the responses of young people along three different contexts: the mental health condition, person using the intervention and technology (Figure 2) to discuss implications for intervention design from the perspective of end-user engagement.

#### 4.2.1. Context I: Mental Health Condition

Developing an understanding of the mental health condition (e.g., common symptoms and related everyday struggles) is crucial to inform the content and format of delivering digital interventions. In terms of content, techniques from Dialectical Behavior Therapy (DBT), Mentalization-Based Therapy (MBT), Therapeutic Assessment (TA), Cognitive Behavioural Therapy (CBT) and improving Self-Compassion could be used to respond to young people’s struggles considering these are the approaches that are most promising in NSSI [40,41] and that some therapeutic principles (e.g., CBT and DBT) have already been successfully translated into digital formats [42,43]. Further, these approaches could also be further translated into more specific interventions adapted to respond to identified needs in this study (e.g., coping with difficult thoughts or emotions).

In terms of format, our study found that in the context of NSSI special efforts need to be focused on supporting young people in the moments of psychological crisis when they experience an urge to self-injure. To achieve this, it is necessary to rethink ways to deliver therapeutic techniques and strategies for this intense state of mind that work quickly and are saliently displayed in a smartphone interface without overburdening the patients with too much information. Current digital interventions are not helpful in emergencies [28], but advancements in Ecological Momentary Assessment (EMA) show that it is possible to assess rapid shifts in mood in patients [44,45] which implies that the future interventions may be better able to deliver support in the moment when young people are most vulnerable.

Similarly, HCI researchers have pointed out that while digital mental health interventions are often delivered in an inflexible content-focused linear way, a more flexible user-driven approach would be better from engagement perspective [26]. To better respond to patients’ mental health needs and increase fit with their everyday life, it is necessary to adapt the delivery of psychological interventions. To illustrate, our study highlighted the need of young people to receive support at different time points (such as before NSSI, after NSSI acts and in preventing relapse) and to receive content adaptive to their everyday life (such as supporting their different coping strategies at home vs. school).

Next, future interventions need to consider *vulnerabilities* related with specific mental health conditions. Young people sometimes expressed their preference to connect with others or create a gallery of their NSSI photos, but some of these ideas may potentially increase NSSI [10]. Because following these preferences could result in harmful interventions, it is necessary to consult with mental health professionals on how to minimize risk related with specific mental health conditions (e.g., avoiding potential triggers in PTSD and not creating a sad music playlist for people with depression). In the case of adolescents’ wish to connect with other young people, intervention developers could thus aim to identify what could be the underlying patients’ needs (e.g., sense of connectedness [46] and find new ways to address them (e.g., through fostering the feeling of common humanity through a self-compassionate mindset intervention [47].

#### 4.2.2. Context II: Person

Adolescents stressed the importance of perceiving the intervention as personally relevant and helpful. Indeed, participants who lack the feeling of personal relevance are more likely to drop out of digital intervention trials [48]. Smartphone apps therefore need to find a way to check their content relevance with their users and explore ways to increase customizability of the interventions beyond simple personalization options.

Delivery of mental health interventions needs to promote users’ choice on engaging with app content and intensity of this engagement. Having choice increases internal motivation according to the self-determination theory [46] and while some app users will need more intensive interventions, others will get better sooner [26].

Importantly, our study suggests that digital resources should consider ways to incorporate young people’s interests in the interventions. Indeed, inserting personal information in an intervention may improve engagement through establishing a sense of ownership [26].

#### 4.2.3. Context III: Technology

Digital interventions need to exploit the benefits of using technology (such as accessibility, flexibility, and novelty) and minimize the limitations (such as long loading times and data security concerns). Existent models, for example the Technology Acceptance Model [49] have found perceived ease of use and perceived usefulness to be two of the most important technology adoption factors. Apps therefore need to minimize possible technological interruptions and assure smooth user experience [50].

Interventions need address young people’s concerns about data security through being transparent about users’ data usage and storage policies of the apps. Indeed, data security and privacy issues fall under critical requirements for technology and engagement [28,51].

Finally, technology provides an exciting opportunity to respond to users’ needs in innovative ways compared to weekly face-to-face psychotherapy. For example, our participants wish to be completely distracted when experiencing the urge to engage in NSSI. App designers could thus explore how to use technology to achieve distraction from intense emotions and only after the users feel calmer present them with mental health interventions. 

### 4.3. Strengths, Limitations and Further Research

This study has limitations that are inherently connected with qualitative approach (e.g., other authors might reach different data interpretations). We addressed this limitation through reflexivity and member checking. The study findings would be strengthened by inclusion of male and/or gender diverse participants. Despite our best efforts, we were not successful in recruiting them. Because more women compared to men engage in NSSI especially in clinical samples [52], our sample is nevertheless representative of the adolescents with NSSI that present to mental health services. We assume that including males and gender diverse people in this study would improve universality of the results. Including such participants might have resulted in similar needs specific to NSSI (e.g., focus on improving thoughts and emotions), however, males might be more likely to indicate the need to manage feelings of anger and/or indicate stronger preferences for inclusion of gamifying elements [53,54].

Using qualitative research allowed us to generate hypotheses that could be tested in the future studies. For example, this study raises important challenges for future digital interventions, such as rethinking delivery of existing interventions in a manner that responds to users’ needs in their everyday lives. The framework developed through a qualitative approach can also serve as a basis for identifying more specific app design requirements through refining our findings in surveys with large sample sizes, co-design workshops and user usability studies. The three contexts in framework also point towards importance of collaboration among HCI researchers (improving user experience and designing technology to improve engagement with treatment), clinicians (sharing their knowledge on mental health context) and finally, people with lived experience who can inform the interventions from the perspective of what works best for them, and how their interests and individual differences could be better represented in developed technology (informing all three contexts).

## 5. Conclusions

This study identified adolescents’ needs and expectations for future mHealth interventions. Based on the results we generated possible directions of designing engaging mental health interventions for patients. We argue that factors related to mental health condition, person and technology will have implications for user-engagement with the developed interventions. To date, research identified that person- and technology-related factors influence engagement with the interventions [24]. Our study adds to this the importance of considering mental health condition as an additional factor that may influence engagement. To illustrate, users who witness improvement in their mental health symptoms may become more engaged in apps and those who are at moments of psychological crisis not supported by the app format may disengage.

Considering the diversity of factors that influence engagement with the future interventions, it is vital that their development profits from collaborations among mental health professionals, HCI professionals and people with lived experience [42,51]. Combination of evidence-based therapeutic approaches and evidence-based design strategies is most promising [55]. Importantly, through collaborating with people with lived experience at various intervention development stages, we can offer interventions that are more personally relevant and engaging.

## Data Availability

The datasets (raw data) are located at the Medical University of Vienna and cannot be shared openly. Requests to access the datasets of the analysed data should be directed to the study authors.
